# Virtual First Emergency Medicine Visits: The Future of Convenient and Efficient Emergency Care

**DOI:** 10.2196/47637

**Published:** 2023-08-03

**Authors:** Matthew Sakumoto

**Affiliations:** 1 University of California, San Francisco San Francisco, CA United States

**Keywords:** telehealth, virtual care, emergency medicine, telemedicine, emergency department, acute care facilities, virtual visit, COVID-19, virtual, utilization, medicine, acute illness, illness, injury, patient, infection, care, physician

## Abstract

The COVID-19 pandemic has led to increased patient volumes, staff shortages, and limited resources in emergency departments, resulting in the rapid acceleration of telemedicine in emergency medicine. The virtual first (VF) program connects patients with emergency medicine clinicians via synchronous virtual video visits, reducing unnecessary emergency department visits and diverting patients to appropriate care settings. VF video visits can improve patient outcomes by providing early intervention for acute care needs and can enhance patient satisfaction by providing convenient, accessible, and personalized care. However, challenges include the lack of physical examination, clinician telehealth training and competencies, and the requirement for a robust telemedicine infrastructure. Additionally, digital health equity is important to ensure equitable access to care. Despite these challenges, the potential benefits of VF video visits in emergency medicine are substantial, and this study is a strong step in building the evidence base for these advancements.

The COVID-19 pandemic has substantially impacted health care delivery worldwide, and emergency medicine is no exception. The pandemic has created unprecedented challenges for emergency departments (EDs), including increased patient volumes, staff shortages, and limited resources. As a result, the use of telemedicine in emergency medicine has been rapidly accelerated, with video visits emerging as a promising alternative to traditional in-person emergency care. This virtual first (VF) model has been used by health systems across the United States and Canada, and prior experiences have demonstrated that on-site ED resources were not needed in the majority of encounters [1-3].

The study “Virtual First Uses Virtual Emergency Medicine Clinicians as a Health System Entry Point: A Cross-Sectional Survey Study” in this issue of the *Journal of Medical Internet Research* investigates the impact of a VF program, which connects patients with emergency medicine clinicians (EMCs) via synchronous virtual video visits, on patients seeking acute care [4]. The program aims to reduce unnecessary ED visits by providing patients with an alternative to seeking in-person care. A survey of VF users showed that 64.2% of respondents would have sought care at urgent care centers or EDs if VF had not been available. Additionally, patients valued the comfort of receiving care at home and the availability of appointments. The authors highlight that EMC-specific expertise and local-regional knowledge are important when they assess the patient’s medical history, perform a physical examination through the video platform, and provide medical advice or refer patients to the appropriate level and location of care, whether it is primary care, urgent care, or emergency care.

One of the primary benefits of VF video visits is the ability to reduce unnecessary ED visits. ED overcrowding has become a substantial problem, and the COVID-19 pandemic has only exacerbated the situation. ED boarding occurs when patients are admitted to the ED but are not transferred to an inpatient bed promptly, leading to longer waiting times, increased patient dissatisfaction, and potential adverse outcomes. VF video visits can help to alleviate ED boarding by diverting patients with minor or nonurgent complaints away from the ED and directing them to appropriate care settings, reducing the burden on EDs [5].

Additionally, VF video visits can improve patient outcomes by providing early intervention for acute care needs. Early intervention is critical in emergency medicine, and VF video visits can facilitate early diagnosis and management of acute conditions, preventing complications and improving patient outcomes. For instance, a patient presenting with chest pain can be triaged and managed through VF, reducing the time to definitive care and potentially saving the patient’s life.

Furthermore, VF video visits have the potential to enhance patient satisfaction by providing convenient, accessible, and personalized care. Patients can access care from the comfort of their homes, avoiding long wait times in crowded EDs. Patients can receive personalized care from EMCs who are specialized in emergency medicine and have access to the patient’s medical records [6]. This lack of interoperability is a barrier faced by many commercial third-party telehealth platforms.

However, VF video visits are not without challenges. One often-cited barrier to VF video visits is the lack of physical examination, which is a crucial component of emergency medicine. EMCs must rely on patients’ self-reported symptoms and history, and video platform limitations may prevent a comprehensive physical examination. This raises the important issue of clinician telehealth training and competencies. How can we better train EMCs to make accurate and safe triage decisions using just the virtual evaluation [7,8]?

Lastly, VF video visits require a robust telemedicine infrastructure, including video platforms, electronic health records, and secure communication channels. These resources can be costly and require substantial investment and maintenance, particularly for smaller health care organizations. On the patient side, we must also be cognizant of digital health equity and make sure that convenient care and potential improved access and cost saving can also be used by underserved and underrepresented populations. Broadband internet and device access, language interpretation services, and the digital literacy to use these tools are all important elements to promote equitable care [9].

Despite these challenges, the potential benefits of VF video visits in emergency medicine are significant. This study is a small but strong step in building the evidence base for these advancements.

## Abbreviations

ED: emergency department
EMC: emergency medicine clinician
VF: virtual first
